# Effects of persimmon peel supplementation on pork quality, palatability, fatty acid composition, and cholesterol level

**DOI:** 10.1186/s40781-016-0114-4

**Published:** 2016-08-22

**Authors:** Sang Moo Lee, Ik Heon Kim, Young Min Choi

**Affiliations:** 1 Department of Animal Sciences, Kyungpook National University, Sangju, 37224 Korea; 2 Sangju Yak-gam Pork, Sangju, 37131 Korea

**Keywords:** Persimmon peel, Pork quality, Sensory quality, Fatty acids, Cholesterol

## Abstract

**Background:**

The objectives of this study were to investigate the effects of persimmon peel (PPM) supplementation on carcass performance, pork quality, eating quality, fatty acid composition, and cholesterol concentration of the porcine *longissimus dorsi* muscle.

**Results:**

No adverse effects of PPM supplementation were observed on carcass and meat quality characteristics among the treatment groups (*P* > 0.05), whereas pork loins from pigs fed a diet supplemented with 0.9 % persimmon peel (T3) showed more tender meat than did pork loins from pigs fed a control diet (*P* < 0.01), even though no significant difference was observed between the control and T1 group. The T3 group had higher ratio of polyunsaturated fatty acids relative to saturated fatty acids (0.33 vs. 0.28, *P* < 0.05) and lower total cholesterol concentration (94.4 vs. 99.1 mg/g, *P* < 0.05) compared to the control group. Persimmon peel appeared to have beneficial effects on fatty acid composition and cholesterol concentration, probably leading to a hypocholesterolemic effect.

**Conclusions:**

Animal diets fortified with persimmon peel represents an efficient and useful method for improving the nutritional quality of pork without impairing growth performance and eating quality properties.

## Background

Persimmon (*Diospyros kaki* Thunb.), a fruit tree originating in China, has been widely cultivated for a long time in Korea, Japan, China, Brazil, and Italy [1]. The pulp of the persimmon fruit is a good source of various important compounds, including tannins, flavonoids, carotenoids, minerals, and vitamins [2, 3]. Due to these functional compounds, persimmon is increasingly drawing attention as a beneficial food for human health; it has long been valued as a folk medicine and as a pharmacological resource in Korea [2–4]. As with persimmon pulp, persimmon peel also possesses numerous functionally diverse compounds, especially polyphenolics [3]. However, persimmon peel is mostly discarded during the drying process in Korean and Japanese production facilities, even though it is a useful resource for humans as a functional food item or additive and in cosmetics [5, 6].

The effects of dietary supplementation with permission peel powder on the perceived quality and cholesterol levels have not been clearly elucidated. The objective of this study was to investigate the effects of persimmon peel, as functional feed additive, on meat quality, sensory quality, fatty acid composition, and cholesterol contents of the porcine *longissimus dorsi* muscle, in order to examine the potential for using an organic waste product as supplementary feedstuff for livestock.

## Methods

### Ethics statement

All animal procedures were approved by the Institutional Animal Care and Use Committee (IACUC) at the Kyungpook National University. All experiments were performed in accordance with the Prevention of Cruelty to Animals Act (1986).

### Animals and muscle samples

A total of 40 crossbred pigs (Landrace × Yorkshire × Duroc; 22 gilts and 18 castrated pigs) with an average initial body weight of 48.9 ± 2.63 kg at 103 d of age were used in this experiment. The pigs were randomly allocated to 4 groups consisting of 10 pigs per group, and all 40 individuals were weighed at the beginning and at the end of the experiment. The environmental conditions to which the pigs were exposed were the same both before and after slaughter. The nutrient levels used for the control and in the treatments were based on standards described in National Research Council [7]. There were no significant differences between the genders or among pig pens in any measurements. Persimmon peel was obtained from persimmon trees growing in Sangju, GyeongBuk, South Korea, and air-dried for a month, then pulverized. The experiment was divided into 4 groups consisting of basal diet (Table 1) with premix supplementation at the level of 0 % (control); 0.3 % persimmon peel powder (Treatment 1; T1); 0.6 % persimmon peel powder (T2); and 0.9 % persimmon peel powder (T3) (Table 2). Each pig was fed *ad libitum*. The major components of persimmon peel powder supplement are presented in Table 3. Persimmon peel powder showed a higher level of total phenolic compound, and was rich in tannin content (24.1 mg/100 g).

**Table 1:** Basal diets composition

Ingredients	Composition (%)
Corn	45.4
Wheat	26.0
Soybean meal	15.0
Wheat Barn	3.7
Molasses	4.5
Animal fat	2.5
Calcium phosphate	1.1
Salt	0.25
Vitamin premix^a^	0.52
Trace mineral premix^b^	0.10
Limestone	0.75
Lysine	0.18
Total	100

^a^Vitamin premix provided per kg: vitamin A, 5500 IU; vitamin D_3_, 1100 IU; vitamin B_2_, 4.4 mg; nicotinic acid, 22 mg; vitamin B_6_, 1.65 mg; vitamin B_12_, 0.22 mg; biotin 0.55 mg; folic acid, 0.33 mg; pantothenic acid 11 mg

^b^Trace mineral premix provided per kg: Cu, 43 mg; Fe, 150 mg; Zn, 55 mg; Mn, 50 mg; Co, 0.5 mg; I, 0.5 mg; Se, 0.2 mg

**Table 2:** Persimmon peel (PP) power composition of the four groups

	Treatment^a^			
	Control	T1	T2	T3
Basal diet^b^ (%)	100.0	99.7	99.4	99.1
PP powder (%)	0.0	0.3	0.6	0.9

^a^Control: basal diet; T1: basal diet with 0.3 % PP powder; T2: basal diet with 0.6 % PP powder; T3: basal diet with 0.9 % PP powder. ^b^Basal diet was in accordance with the National Research Council (1998)

**Table 3:** Major components of persimmon peel (PP) powder

Traits	PP powder
Vitamin A (mg/100 g)	42.25
Vitamin C (mg/100 g)	46.35
TPC (mg/100 g)	44.07
Ca (%)	0.01
Mg (%)	0.05
K (%)	1.27
Na (%)	0.03
P (%)	0.09

*Abbreviation*: *TPC* total phenolic compound

All pigs were transported to a commercial abattoir under the same handling conditions at the same age (188 d of age; 135 ± 3.4 kg), and slaughtered by electric stunning during the autumnal period under the supervision of the Korean grading service for animal products. All animals were exsanguinated and placed in a dehairer at 65 °C for 5 min, with remaining hair removed with a flame and knife after exiting from the dehairer. Carcasses were weighed following evisceration. The mean of the loin eye area (measured at the level of the last rib) and back-fat thickness (measured at the 11th and last thoracic vertebrae) was used.

At 45 min postmortem, muscle samples were taken from the *longissimus dorsi* muscles at the 7th and 8th thoracic vertebrae to measure muscle pH (pH_45 min_). After 24 h postmortem in a 4 °C cold room, pork loins were collected for meat quality measurements, and samples were frozen and stored at −20 °C for later analysis of sensory characteristics, fatty acid composition, and cholesterol content of cooked pork.

### Meat quality characteristics

In order to estimate the postmortem glycolytic rate, muscle pH at 45 min (pH_45 min_) and 24 h (pH_24 h_) postmortem were measured directly by piercing the carcasses at the 8th and 9th thoracic vertebrae with a spear-type portable pH meter (IQ-150 pH meter and PH77-SS probe, IQ Scientific Instruments Inc., San Diego, CA).

At 24 h postmortem, meat color was measured with a Minolta chromometer (CR-400, Minolta Camera Co., Japan) following surface exposure for 30 min at 4 °C. Results were expressed as Commission Internationale de l’Eclairage [8] lightness (L^*^), redness (a^*^), and yellowness (b^*^) values, with the average of three measurements used. Drip loss, filter-paper fluid uptake (FFU), and cooking loss were measured to assess water-holding capacity (WHC). To determine drip loss, samples weighing approximately 80 g were trimmed and accurately weighed. Each sample was then placed in a square netting (25 cm^2^) and then suspended in an inflated plastic bag for 48 h at 4 °C, ensuring that the sample did not make contact with the bag, after which they were re-weighed [9], with drip loss calculated as the percentage change in weight [9]. For FFU and cooking loss, pork loins were cut into 2-cm thick chops. To measure FFU, a filter paper (Whatman #2, 42.5 mm in diameter) was pre-weighed, placed on the surface of a chop for less than 2 s to absorb its fluids, and then weighed again; FFU was expressed as mg of exudates absorbed into the filter paper [10]. To measure cooking loss, the samples were put in thin-walled polyethylene bags and placed in a water bath (80 °C) until the internal temperature (measured using a thermometer with a handled probe; TES-1300, TES Electrical Electronic Co., Taiwan) reached 71 °C. Pork samples were then cooled in ice water for 15 min. The cooled samples were taken from the polyethylene bag, blotted dry, and weighed. Cooking loss was determined by weighing the chops before and after cooking [9]. Preparation of cooked meat samples for Warner–Bratzler shear force (WBS) was similar to the procedure for cooking loss, and WBS was performed with six to ten cores (1.27 cm in diameter) removed from the steak parallel to the longitudinal orientation of the muscle fibers. WBS was determined using an Instron Universal Testing Machine (Model 1011, Instron Corp., USA) equipped with a Warner–Bratzler shearing device.

Color and marbling were evaluated using fresh pork loin at 24 h postmortem based on the National Pork Producer Council (NPPC) color and marbling standards [11]. Twenty-mm thick pork loin chops were exposed to air for 30 min at 4 °C to bloom. Color scores ranged from 1 (pale) to 6 (dark), and marbling score ranged from 1 (1 % intramuscular fat [IMF] content) to 10 (10 % IMF content).

### Eating quality evaluation

A sensory panel consisting of 12 pork-consuming individuals (20–40 years of age, 6 females and 6 males) was employed to evaluate the sensory attributes of the cooked pork. Panelists were selected based on their interest and availability. All training and testing was conducted at Kyungpook National University. Before sensory evaluation, all panelists were trained for at least 6 months (3 times per wk) and for up to 1 h in each training session. Panelist training was performed in accordance with practices described by the American Meat Science Association [12] and previously published procedures [13]. All pork samples were evaluated twice.

Samples were thawed overnight at 4 °C and then cooked in a humid heat oven (MCS312CF4, Electrolux, Sweden, set at 180 °C) to an internal temperature of 71 °C, as measured by a TES-1300 thermometer (TES Electrical Electronic Co., Taiwan). Samples were then immediately sliced into 1.3 cm × 1.3 cm × 1.3 cm pieces, which were subsequently randomly selected to minimize bias. The chosen samples were placed into 1-ounce lidded glass jars labeled with random three-digit codes and kept in a water bath (54 °C) until presented to the panelists. All samples were presented to panelists simultaneously on a compartmented plate, with an interval of approximately 5 min between evaluations of consecutive samples. For sensory evaluation, panelists were seated in private booths under incandescent light, and served distilled water (at room temperature) and salt-free crackers before the first sample and between samples to cleanse their palate.

Cooked samples were evaluated for softness (force required to compress the meat sample between the molar teeth; 1 = very hard; 9 = very soft), initial tenderness (force required to chew 3 times after initial compression; 1 = very tough; 9 = very tender), chewiness (energy required at the 9th chew to swallow at a constant rate; 1 = very chewy; 9 = very tender), rate of breakdown (number of chews required for the sample to disintegrate during the mastication process in preparation for swallowing; 1 = very slow; 9 = very fast), mouth coating (amount of oil/fat left in the mouth surface; 1 = none; 9 = very high), amount of perceptible residue (amount of connective tissue remaining upon complete disintegration of meat sample; 1 = abundant; 9 = none), juiciness (amount of moisture released after 5 chews; 1 = not juicy; 9 = extremely juicy), flavor intensity (intensity of pork flavor after 8 chews; 1 = no pork flavor; 9 = full pork flavor), and off-flavor intensity (intensity of any flavor or after-taste perceived as inappropriate to cooked pork; 1 = very strong; 9 = very weak) [12, 13].

### IMF content, fatty acid composition, and cholesterol content

The content of IMF was determined using the Soxhlet method with a solvent extraction system [14]; fat was extracted based on the method described by Folch et al. [15]. Briefly, 1.5 g of homogenized meat was twice blended with an extraction solvent of chloroform/methanol (2:1, v/v), filtered, and then placed in a separator funnel and mixed with saline solution (0.9 % NaCl). After separation into two phases, the aqueous methanol fraction was discarded, whereas the chloroform lipid fraction was washed with extraction solvent. Following further filtration and evaporation by means of a rotary evaporator, the lipid extracts were transferred to test-tubes for subsequent gas chromatographic analysis. Duplicates of lipids were methylated by adding 2 ml of sodium methoxide, distilled water, and heptane. Gas chromatograph analysis was performed on a Gas Chromatography–Mass Selective Detector (GC, Agilent 7890 N, USA; MSD, Agilent 5975A, USA) equipped with a HP-INNOWAX column (length 30 m, internal diameter 0.25 mm, film thickness 0.25 μm). The operating conditions consisted of a helium flow rate of 0.7 ml/min, the FID detector set to 260 °C, the split-splitless injector set to 220 °C with an injection rate of 120 ml/min, and an injection volume of 1 μl. The temperature program of the column consisted of 4 min at 140 °C followed by a subsequent increase to 220 °C at a rate of 4 °C/min. The retention time and area of each peak were computed using Agilent software. The individual fatty acid peaks were identified by comparing the retention times with those of known mixtures of standard fatty acids (FAME, Sigma-Aldrich CO., USA). Fatty acid composition was expressed as the percentage of total methylated fatty acid. The hypocholesterolemic/hypercholesterolemic potential of the pork samples was calculated according to procedures set out by Santos-Silva et al. [16] using the *h/H* index [h/H = (18:1n-9 + 18:2n-6 + 20:4n-6 + 18:3n-3 + 20:5n-3 + 22:5n-3 + 22:6n-3)/(14:0 + 16:0)].

Concentration of total and low-density lipoprotein (LDL) cholesterol in muscle tissues were determined with enzymatic method using a Bio Merieux kit (Bio-Meraux Lab, Paris, France), and concentrations of high-density lipoprotein (HDL) and total cholesterol in muscle tissues were measured using Alpha Diagnostics kit (Alpha Diagnostics Inc., CA, USA).

### Statistical analyses

Statistical analyses were performed using SAS software [17]. Analyses of variance were conducted to evaluate potential differences (*P* < 0.05) between the different treatments. The results are presented as means for each group along with their standard deviations.

## Results

### Carcass performance, meat quality, and palatability

Carcass characteristics of the control and treatment groups are presented in Table 4. There were no significant differences in carcass weight, loin eye area, and back-fat thickness among the treatments (*P* > 0.05). The effects of persimmon peel supplementation on meat quality characteristics are shown in Table 5. There were no significant differences in the postmortem glycolytic rate, including muscle pH_45 min_ and pH_24 h_, among the groups (*P* > 0.05). Lightness (48.1 and 47.1, *P* > 0.05), drip loss (2.68 and 2.47 %, *P* > 0.05), and cooking loss (22.8 and 22.5 %, *P* > 0.05) were also not significantly different between the control and T3 groups, respectively. No significant difference was also observed between the control and T3 groups in FFU, even though pork samples from group T3 had a significantly lower FFU than that of samples from group T1 (29.0 vs. 69.6 mg, *P* < 0.05). WBS as an indicator of tenderness was lower in the T3 group than in the control group (39.7 vs. 48.9 N, *P* < 0.05). Neither NPPC color nor marbling scores were affected by dietary supplementation (*P* > 0.05).

**Table 4:** Effects of persimmon peel (PP) supplementation on carcass performance and mineral contents in porcine *longissimus dorsi* muscle

	Treatment^a^				Level of significance
	Control	T1	T2	T3	
Carcass performance					
Carcass weight (kg)	86.3 ± 9.87	93.6 ± 4.93	90.6 ± 11.4	92.0 ± 7.55	NS
Loin eye area (cm^2^)	66.7 ± 1.35	67.0 ± 7.74	62.7 ± 6.82	67.8 ± 8.78	NS
Back-fat thickness (mm)	20.3 ± 2.52	22.0 ± 5.29	26.3 ± 6.11	24.6 ± 8.74	NS

^a^Control: basal diet; T1: basal diet with 0.3 % PP powder; T2: basal diet with 0.6 % PP powder; T3: basal diet with 0.9 % PP powder

Results are expressed in mean value ± SD

Levels of significance: NS = not significant

**Table 5:** Effects of persimmon peel (PP) supplementation on meat quality characteristics of porcine *longissimus dorsi* muscle

	Treatment^c^				Level of significance
	Control	T1	T2	T3	
Muscle pH_45 min_	5.85 ± 0.07	5.91 ± 0.20	5.91 ± 0.07	5.86 ± 0.13	NS
Muscle pH_24 h_	5.35 ± 0.10	5.41 ± 0.09	5.46 ± 0.16	5.50 ± 0.07	NS
Lightness (*L* ^*^)	48.1 ± 1.04	47.4 ± 1.30	46.2 ± 1.44	47.1 ± 1.69	NS
Redness (*a* ^*^)	4.11 ± 0.98	4.55 ± 1.12	4.07 ± 1.18	4.38 ± 0.88	NS
Yellowness (*b* ^*^)	4.53 ± 0.80	4.32 ± 0.81	3.72 ± 0.32	4.15 ± 0.71	NS
Drip loss (%)	2.68 ± 1.27	3.89 ± 1.25	4.45 ± 1.14	2.47 ± 0.96	NS
FFU (mg)	36.7 ± 7.81^b^	69.6 ± 22.5^a^	54.8 ± 11.9^ab^	29.0 ± 3.51^b^	*
Cooking loss (%)	22.8 ± 4.94	19.1 ± 1.44	22.1 ± 2.79	22.5 ± 4.12	NS
WBS (N)	48.9 ± 2.27^a^	43.0 ± 3.69^ab^	43.2 ± 2.66^ab^	39.7 ± 2.56^b^	*
NPPC color	2.33 ± 0.29	3.00 ± 1.32	2.25 ± 0.66	2.33 ± 0.58	NS
NPPC marbling	2.33 ± 0.76	3.00 ± 1.32	2.58 ± 0.14	2.50 ± 1.00	NS

^c^Control: basal diet; T1: basal diet with 0.3 % PP powder; T2: basal diet with 0.6 % PP powder; T3: basal diet with 0.9 % PP powder

Results are expressed in mean value ± SD

Levels of significance: NS = not significant; *P < 0.05

^a-b^Means within a row with different superscripts differ significantly (*P* < 0.05)

*Abbreviations*: *FFU* filter-paper fluid uptake, *WBS* Warner Bratzler shear force, *NPPC* National Pork Producers Council

Eating quality characteristics of pork loin for each group classified by the supplemental levels of persimmon peel are presented in Table 6. As with the instrumental tenderness parameters, a few tenderness attributes were found to be significantly different among the treatment groups. Pork samples from group T3 had higher values for softness (6.54 vs. 5.07, *P* < 0.05) and initial tenderness (6.20 vs. 4.82, *P* < 0.05) compared to samples from the control group, respectively, whereas softness (5.51, *P* > 0.05) and initial tenderness (5.19, *P* > 0.05) values of the T1 group were similar to those of the control group. There were no significant differences among the treatment groups (*P* > 0.05) for other tenderness attributes, including chewiness, rate of breakdown, mouth coating, and amount of perceptible residue; moreover, there were no significant difference in juiciness and flavor intensity among the treatment groups (*P* > 0.05).

**Table 6:** Effects of persimmon peel (PP) supplementation on sensory quality characteristics of porcine *longissimus dorsi* muscle

	Treatment^d^				Level of significance
	Control	T1	T2	T3	
Softness	5.07 ± 0.26^c^	5.51 ± 0.18^bc^	6.03 ± 0.57^ab^	6.54 ± 0.14^a^	*
Initial tenderness	4.82 ± 0.45^c^	5.19 ± 0.08^bc^	5.81 ± 0.08^ab^	6.20 ± 0.28^a^	**
Chewiness	5.75 ± 1.98	6.01 ± 0.98	5.65 ± 0.98	6.36 ± 0.22	NS
Rate of breakdown	5.54 ± 1.46	6.07 ± 0.57	5.05 ± 0.49	5.81 ± 0.13	NS
Mouth coating	3.67 ± 0.44	4.12 ± 0.76	3.63 ± 0.22	3.70 ± 0.22	NS
Amount of perceptible residue	5.85 ± 0.46	5.75 ± 0.33	5.01 ± 0.46	5.17 ± 0.47	NS
Juiciness	5.29 ± 0.83	5.33 ± 0.49	5.23 ± 0.20	5.54 ± 0.29	NS
Flavor intensity	6.30 ± 0.29	6.31 ± 0.27	5.66 ± 0.39	6.15 ± 0.69	NS
Off flavor intensity	5.96 ± 0.47	6.23 ± 0.22	6.13 ± 0.46	5.81 ± 0.19	NS

^d^Control: basal diet; T1: basal diet with 0.3 % PP powder; T2: basal diet with 0.6 % PP powder; T3: basal diet with 0.9 % PP powder

Levels of significance: NS = not significant; *P < 0.05; **P < 0.01

^a-c^Means within a row with different superscripts differ significantly (*P* < 0.05)

Score distribution: low to high, softness: hard to soft; initial tenderness: tough to tender; chewiness: very chewy to very tender; rate of breakdown: very slow to very fast; mouth coating: none to very high; amount of perceptible residue: abundant to none; juiciness: not juicy to extremely juicy; flavor intensity: very weak to very strong; off-flavor intensity: very strong to very weak

### IMF, fatty acid composition, and cholesterol content

There were marked differences in fatty acid composition and cholesterol levels (Table 7), even though no significant differences were observed in IMF among the treatment groups (*P* > 0.05). Compared with pigs fed the control diet, pork from pigs fed T3 diets had a lower percentage of saturated fatty acid (SFA, 41.5 vs. 43.7 %, *P* < 0.05) and higher percentage of polyunsaturated FA (PUFA, 13.6 vs. 12.1 %, *P* < 0.05). The lower SFA in the T3 group was most probably caused by differences in the composition of palmitic acid (16:0); the percentage of palmitic acid was higher in the control group than in the T3 group (26.95 vs. 23.9 %, *P* < 0.01; data not shown). There were no significant differences in SFA (43.2 %, *P* > 0.05), monounsaturated FA (MUFA, 44.1 vs. 43.7 %, *P* > 0.05), n-6 (11.6 vs. 12.4 %, *P* > 0.05), and n-3 (0.57 vs. 0.67 %, *P* > 0.05) between the control and T2 groups. The *h/H* index of the T3 group was the highest compared to the other groups (2.09, *P* < 0.01), and no significant difference was observed between the control and T1 groups (1.78 vs. 1.83, *P* > 0.05). Pork samples from group T3 had lower concentrations of total cholesterol (94.4 vs. 99.1 mg/100 g, *P* < 0.05) and LDL cholesterol (47.4 vs. 51.4 mg/100 g, *P* < 0.05) than those of the control group, whereas no significant differences were observed in the concentration of total cholesterol between the control and T1 groups (96.4 mg/100 g, *P* > 0.05).

**Table 7:** Effects of persimmon peel (PP) supplementation on intramuscular fat (IMF), fatty acid composition, and cholesterol content of porcine *longissimus dorsi* muscle

	Treatment^c^				Level of significance
	Control	T1	T2	T3	
IMF (%)	1.90 ± 0.80	2.90 ± 1.22	2.66 ± 0.23	2.78 ± 0.99	NS
Fatty acids					
SFA (%)	43.7 ± 0.35^a^	43.6 ± 0.61^a^	43.2 ± 0.59^a^	41.5 ± 0.81^b^	*
MUFA (%)	44.1 ± 0.63	44.4 ± 1.01	43.7 ± 0.80	45.0 ± 1.22	NS
PUFA (%)	12.1 ± 0.21^b^	12.0 ± 0.45^b^	13.1 ± 0.55^a^	13.6 ± 0.65^a^	*
PUFA:SFA	0.28 ± 0.02^b^	0.28 ± 0.02^b^	0.30 ± 0.01^ab^	0.33 ± 0.01^a^	*
n-6 (%)	11.6 ± 0.97	11.4 ± 1.79	12.4 ± 1.51	12.9 ± 0.41	NS
n-3 (%)	0.57 ± 0.06	0.58 ± 0.10	0.67 ± 0.14	0.68 ± 0.15	NS
n-6:n-3	20.3 ± 1.31	19.7 ± 1.37	18.7 ± 1.93	19.6 ± 4.61	NS
*h/H* index	1.78 ± 0.07^b^	1.83 ± 0.07^b^	1.91 ± 0.11^b^	2.09 ± 0.12^a^	**
Cholesterol (mg/100 g)					
Total cholesterol	99.1 ± 1.46^a^	96.4 ± 0.97^ab^	98.1 ± 2.60^ab^	94.4 ± 2.46^b^	*
HDL cholesterol	25.3 ± 1.23	29.4 ± 3.60	29.2 ± 0.39	25.5 ± 1.61	NS
LDL cholesterol	51.4 ± 1.40^a^	45.2 ± 3.13^b^	46.6 ± 1.40^b^	47.4 ± 1.52^b^	*

^c^Control: basal diet; T1: basal diet with 0.3 % PP powder; T2: basal diet with 0.6 % PP powder; T3: basal diet with 0.9 % PP powder

Results are expressed in mean value ± SD

Levels of significance: NS = not significant; *P < 0.05; **P < 0.01

^a-b^Means within a row with different superscripts differ significantly (*P* < 0.05)

*Abbreviations*: *SFA* saturated fatty acid, *MUFA* monounsaturated fatty acid, *PUFA* polyunsaturated fatty acid, *n-6* sum of linoleic (C18:2n-6) γ-linolenic (C18:3n-6), eicosadienoic (C20:2n-6), and arachidonic (C20:4n-6) acids, *n-3* sum of α-linolenic acid (C18:3n-3), eicosatrienoic acid (C20:3n-3), EPA (C20:5n-3), and DHA (C22:6n-3), *n-6:n-3* ratio of n-6 to n-3, *h:H index* ratio of hypocholesterolemic to hypercholesterolemic potential [h/H = (18:1n-9 + 18:2n-6 + 20:4n-6 + 18:3n-3 + 20:5n-3 + 22:5n-3 + 22:6n-3)/(14:0 + 16:0)], *HDL* high-density lipoprotein, *LDL* low-density lipoprotein

## Discussion

Oriental persimmon peel has a high concentration of sugars and nutritional antioxidant vitamins [2, 3], and contains many biological polyphenols, such as tannins, flavonoids, and carotenoids, which have a scavenging action against active oxygen free radicals [1]. Thus, persimmon peel and its polyphenolics impart benefits to animal health [1, 3] without impairing growth performance and meat quality characteristics [18]. The results from the current study support this notion, in that in this study, no adverse effects of persimmon peel supplementation were observed on carcass characteristics and meat-quality characteristics. In the WHC, drip loss values are generally correlated with FFU values [10]. In this study, even though FFU was significantly higher in the T1 group compared to the control group (*P* < 0.05), no significant difference was observed the control and T1 groups (2.68 vs. 3.89 %, *P* > 0.05) due to higher standard deviations. However, the meat from pigs of all of the treatment groups were described as reddish-pink, firm, and non-exudative pork, which quality pork is classified based on pH_45 min_ (>5.8), drip loss (2–6 %), and lightness (42–50) values [19–22]. In addition, instrumental tenderness parameters (*P* < 0.05) and initial tenderness measured by trained panelists (*P* < 0.01) were significantly lower in pork loins from group T3, indicating more tender meat compared to samples from the control group.

Meat products are a good source of various fatty acids in the human diet [23]. As fatty acid balance is more important than the percentage of each fatty acid individually, World Health Organization/Food and Agriculture Organization (WHO/FAO) experts have suggested guidelines for a balanced diet to prevent heart disease, with the recommended ratio of PUFA:SFA above 0.4 [24]. In the current study, although the PUFA:SFA ratio of the control and treatment groups were lower than the WHO/FAO recommended value, the T3 group exhibited a higher ratio of PUFA:SFA (0.33 vs. 0.28, *P* < 0.05) and a lower percentage of palmitic acid than that of the control group. Moreover, the ratio of PUFA:SFA was similar between the control and T2 groups (0.30, *P* > 0.05), a result associated with persimmon peel supplementation. Gorinstein et al. [25] suggested that dietary persimmon pulp and peel can improve lipid metabolism, and rats fed diets containing persimmon had a lower percentages of SFA including palmitic acid than did rats fed diets without persimmon due to the increase antioxidant activity [26].

Excessive consumption of cholesterols, especially LDL cholesterol, and a high-fat diet can increase lipid accumulation in the blood and damage blood-vessel walls, a condition associated with atherosclerosis, hypertension, and heart stroke [27]. Clinical investigations have shown that natural antioxidants and polyphenolic compounds can reduce the risk of cardiovascular disease [28], and significantly lower plasma LDL cholesterol was observed in rats fed with persimmon peel than in rats fed a basal diet [25]. Moreover, Son et al. [6] reported that persimmon peel had an inhibitory effect on aortic vessel thickening, suggesting anti-atherogenic effects, which the results from the current study support; in this study, the hypocholesterolemic potential (*h/H* index) was higher in the T3 group than in the control group (*P* < 0.01). A statistically significant decrease in total (*P* < 0.05) and LDL (*P* < 0.05) cholesterol concentrations were observed in pigs fed a diet supplemented with 0.9 % persimmon peel supplementation compared to pigs fed the control diet.

## Conclusions

Our results indicated that persimmon peel supplementation had beneficial effects on fatty acid composition and cholesterol concentration, likely leading to a hypocholesterolemic effect, even though no practical effects on carcass and meat-quality characteristics were observed. Thus, we conclude that fortifying diets with persimmon peel supplements represents an efficient and effective method for improving the nutritional quality of pork without impairing growth performance and eating quality.

## Abbreviations

FAO, food and agriculture organization; FFU, filter-paper fluid uptake; h/H, hypocholesterolemic/hypercholesterolemic; HDL, high-density lipoprotein; IMF, intramuscular fat; LDL, low-density lipoprotein; NPPC, national pork producer council; PPM, persimmon peel; PUFA, polyunsaturated fatty acid; SFA, saturated fatty acid; WBS, Warner-Bratzler shear force; WHO, world health organization
